# MiSynPat: An integrated knowledge base linking clinical, genetic, and structural data for disease‐causing mutations in human mitochondrial aminoacyl‐tRNA synthetases

**DOI:** 10.1002/humu.23277

**Published:** 2017-06-27

**Authors:** Luc Moulinier, Raymond Ripp, Gaston Castillo, Olivier Poch, Marie Sissler

**Affiliations:** ^1^ CSTB Complex Systems and Translational Bioinformatics ICube Laboratory and Strasbourg Federation of Translational Medicine (FMTS) CNRS Université de Strasbourg Strasbourg France; ^2^ Université de Strasbourg CNRS Architecture et Réactivité de l'ARN Strasbourg France

**Keywords:** 3D structures, aminoacyl‐tRNA synthetases, disease‐causing mutations, knowledge base, mitochondrial disorders, sequence alignments

## Abstract

Numerous mutations in each of the mitochondrial aminoacyl‐tRNA synthetases (aaRSs) have been implicated in human diseases. The mutations are autosomal and recessive and lead mainly to neurological disorders, although with pleiotropic effects. The processes and interactions that drive the etiology of the disorders associated with mitochondrial aaRSs (mt‐aaRSs) are far from understood. The complexity of the clinical, genetic, and structural data requires concerted, interdisciplinary efforts to understand the molecular biology of these disorders. Toward this goal, we designed MiSynPat, a comprehensive knowledge base together with an ergonomic Web server designed to organize and access all pertinent information (sequences, multiple sequence alignments, structures, disease descriptions, mutation characteristics, original literature) on the disease‐linked human mt‐aaRSs. With MiSynPat, a user can also evaluate the impact of a possible mutation on sequence‐conservation‐structure in order to foster the links between basic and clinical researchers and to facilitate future diagnosis. The proposed integrated view, coupled with research on disease‐related mt‐aaRSs, will help to reveal new functions for these enzymes and to open new vistas in the molecular biology of the cell. The purpose of MiSynPat, freely available at http://misynpat.org, is to constitute a reference and a converging resource for scientists and clinicians.

## 1 INTRODUCTION

Aminoacyl‐tRNA synthetases (aaRSs) contribute critically to protein biosynthesis by catalyzing the specific ligation of amino acids onto their cognate tRNA(s). In human mitochondria, the translation machinery is devoted to the synthesis of 13 proteins, all subunits of the respiratory chain complexes. Mitochondrial (mt) aaRSs are thus implicated in cellular energy (ATP) production. Except for GlyRS and LysRS that are encoded by single genes, human mt‐aaRSs are encoded in the nucleus by a set of genes distinct from those coding for the cytosolic aaRSs (Bonnefond et al., 2005). Mt‐aaRSs are all synthesized in the cytosol, addressed to and imported into the mitochondria, thanks to the presence of an N‐terminal pre‐sequence (MTS, for “mitochondrial targeting sequence”), which is presumably cleaved upon entry to mitochondria (Carapito et al., 2017).

The first correlation between a mutation affecting a mt‐aaRS and a human disease dates back to 2007, when mutations within the *DARS2* gene, coding for mt‐AspRS, were associated with a leukoencephalopathy (LBSL) (Scheper et al., 2007). This first description attracted the attention of the medical community, and many other disease‐causing mutations have been discovered since. Today, all 19 mt‐aaRS‐encoding genes have been reported to be affected (reviewed in e.g. Diodato, Ghezzi, & Tiranti, 2014; Konovalova & Tyynismaa, 2013; Oprescu, Griffin, Beg, & Antonellis, 2017; Schwenzer, Zoll, Florentz, & Sissler, 2014; Suzuki, Nagao, & Suzuki, 2011). Except for GlyRS and LysRS, which present dominant mutations, all mutations lead to autosomal recessive disorders, with patients being either homozygotes or compound heterozygotes. Despite being ubiquitously expressed and probably having a common role in a single cellular process, that is mt translation, mt‐aaRSs are impacted in various ways. Their mutations cause pleiotropic effects with an unexpected variety of phenotypic expressions, including mainly neurological disorders but also non‐neurological symptoms. Besides the fact that new mutations are continuously discovered, neither the cause of the selective vulnerability nor the molecular mechanisms leading to the diseases are well understood.

It is therefore timely to extract and collect existing and emerging data related to mutations affecting human mt‐aaRSs to allow the community of clinicians and fundamental researchers to analyze them comprehensively and systematically within a dedicated and continuously updated computing infrastructure. To achieve this, we developed **MiSynPat** (for **Mi**tochondrial aminoacyl‐tRNA **Syn**thetases and **Pat**hologies), a knowledge database and a Web server, for the characterization, analysis, and querying of the interrelations between genetics, mutations, sequences, 3D structures, multiple alignments, sequence/structure conservation, and biological functional domains. The architecture of MySinPat allows to search for data *via* four main interconnected information levels focused on the disease‐causing mutations: protein sequence alignments, 3D structures, literature, and statistics. To ensure the quality, robustness, and durability of MiSynPat, helper modules have been designed to semi‐automatically collect and integrate newly published data or literature. MiSynPat interface is intentionally designed to be user‐friendly and accessible to a broad audience of both specialists (clinicians and researchers) and non‐specialists (e.g., patients, patient associations, etc.).

## 2 DESCRIPTION OF MiSynPat COMPONENTS AND TOOLS

The MiSynPat infrastructure is organized around three main components: (i) a knowledge base; (ii) an updating system for new 3D structure and literature information with access restricted to authorized experts; (iii) a Web interface to query the database.

The knowledge base is organized around four main SQL tables and two junction tables (see *Materials and Methods* and Supp. Fig. S1), which provide the list of mutations and related information, information on protein sequences, homologs and related 3D structures as well as links to the original bibliography and associated diseases. Each mutation is characterized at the disease, genetic, evolutionary, and structural levels and an access is provided to the statistics calculated for individual or all synthetases. Currently, the MiSynPat knowledge base contains:

- Nineteen multiple sequence alignments with a total of 1,764 sequences from divergent bacterial, archaeal, and eukaryotic species, including the sequences from 100 PDB entries (Protein Data Bank, http://www.rcsb.org). For eukaryotic species, organellar or cytosolic aaRSs have been distinguished. In addition, the 19 multiple sequence alignments of the mt‐aaRSs UniProt Reference sequences from 58 divergent vertebrates are provided.
- 3D coordinates for the three available crystallographic structures of human mt‐aaRSs and for the models obtained by automated homology modeling of the 19 mt‐aaRSs at a PHYRE2 confidence level >90% for all systems.
- Two hundred and eight disease‐related variations with the allelic composition of each patient and their correlation with the eight distinct major disorders.
- One hundred and twenty‐five abstracts and PMIDs linked to the NCBI PubMed database for the publications retained by the double sieve updating system (see below).

A set of helper tools (JSmol, Modeler, LSQMan, NAccess, PolyPhen, SIFT) is attached to the knowledge base to provide and manage the data. In addition, in‐house statistical packages and updating systems have been developed.

### 2.1 Updating system

The quality and lifetime of a knowledge‐based Web server strongly depend on the balance between maximum automation for its update and minimum human dependency. To incorporate new bibliographic information into the database, the chosen strategy is based on a double sieve mechanism. Firstly, publications concerning human mt‐aaRS diseases are automatically retrieved from PubMed through the NCBI Web service using a specialized filter. Secondly, the articles selected by the first sieve are presented through a Web form to an expert who validates or rejects them and enters the relevant information regarding mutations/diseases into the database. Missense, nonsense, insertion, deletion, and splicing defects present in the mt‐aaRS exons or introns are all registered and those impacting the protein sequence are further visualized. The bibliography search and validation process is performed daily and has been tested over a period of 18 months. After training and adjustment of the double sieve system, two‐thirds of the newly presented publications were relevant.

### 2.2 Web interface

**Figure 1:**
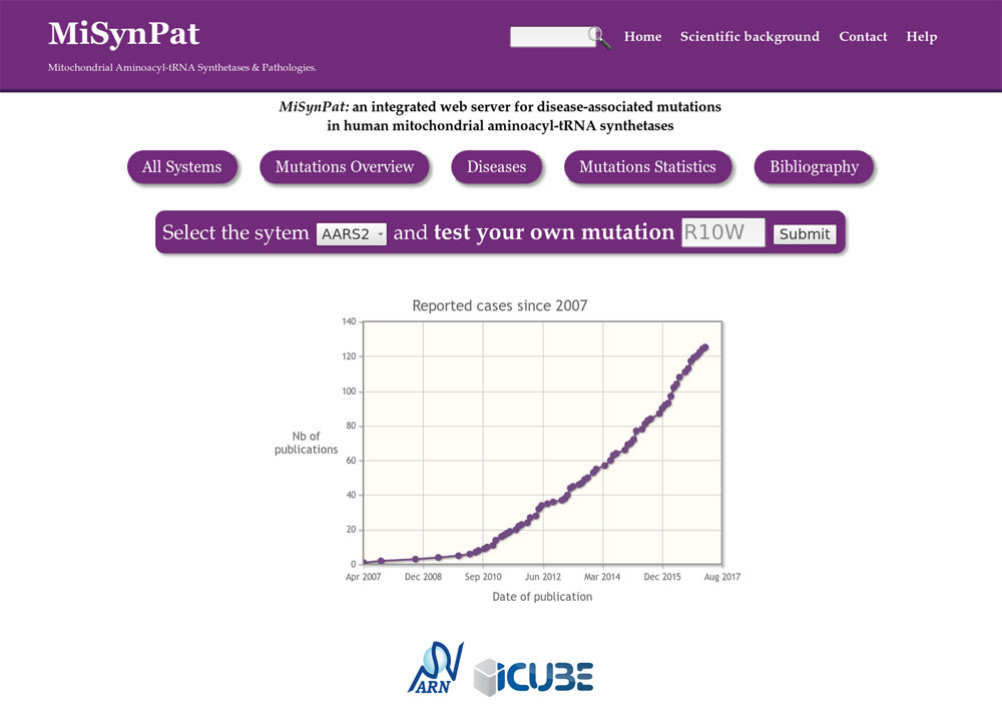
Screen capture of the Home page. The home page provides access to all MiSynPat resources. Four main sections have been designed. The “header” section has a text search widget and access to the home page as well as to a synthetic description of the mt‐aaRSs scientific background, the contact and the help pages. The “access” section has five main entry points: All systems, Mutations Overview, Diseases, Mutations Statistics, and Bibliography. The “mutation” section gives direct access to the mutation modeling tool and the “bibliography” section provides a plot of the automatically updated cumulative number of bibliographic entries with reported cases of human mt‐aaRS disorders is shown in an automatically updated plot

The *home page* at http://misynpat.org (Fig. 1) provides access to all MiSynPat resources. To provide a global overview and an intuitive navigation, five main entry points are accessible (All systems, Mutations Overview, Diseases, Mutations Statistics, and Bibliography) combined with a direct access to the mutation modeling tool. In addition, the cumulative number of bibliographic entries with reported cases of human mt‐aaRS disorders is shown in an automatically updated plot.

**Figure 2:**
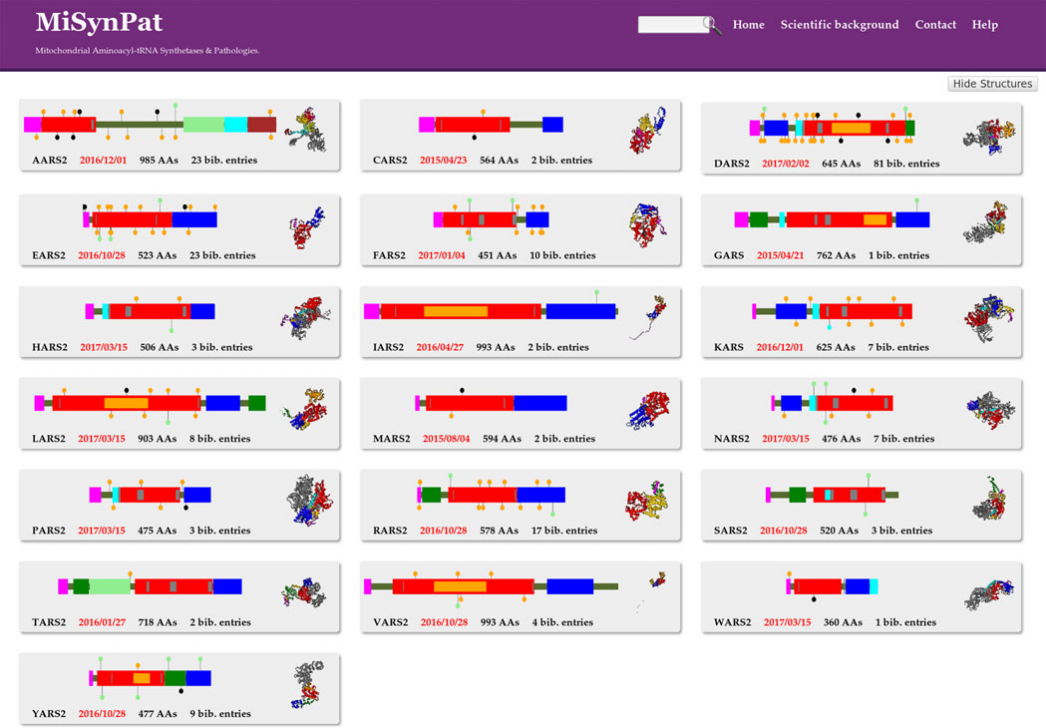
Screen capture of the “All Systems” synoptic page. The page provides general overviews of the 19 interactive insets corresponding to the 19 human mt‐aaRSs. Each inset contains the ModMutGimmick with the mutations localized by lollipops, the 3D structure thumbnail, the name and length of the human mt‐aaRS, the date of the most recent update and the current number of bibliographic entries (bib. entries). The ModMutGimmick is as an interactive true‐scale graphical representation of the mt‐aaRS modular organization with colored boxes (see Supp. Table S3) indicating idiosyncratic and common functional regions and motifs. The reported mutations are schematized as follow: (i) recessive disease‐related missense mutations are indicated by green and orange lollipops for homozygous and compound heterozygous mutations, respectively, (ii) dominant mutations are indicated by cyan lollipops, and (iii) nonsense mutations are indicated by black lollipops

- The “All systems” synoptic page displays the 19 interactive insets corresponding to the 19 human mt‐aaRSs. Each inset is clickable to access the single mt‐aaRS window (see below) and includes an image of the modular and mutational schema of the considered mt‐aaRS (hereafter called ModMutGimmick), its 3D structure thumbnail, its name, the date of the last change in the information content of the considered mt‐aaRS, the length of the human protein sequence and the current number of bibliographic entries per system (Fig. 2). Of note, no inset for mt‐GlnRS is provided since there is no mt‐GlnRS gene and it has been demonstrated that Gln‐tRNA^Gln^ is produced *via* an alternate indirect pathway (Echevarría et al., 2014).
- The “Mutations Overview” page gives access to the exhaustive list of clinical cases, with indications of the two variants in allelic compositions, the related disorders (with OMIM access if applicable), the publication years and PubMed links to relevant publications. The list can be sorted according to each item and can be queried through a text search widget.
- The “Diseases” page lists, if applicable, the abbreviated and complete disease name, the OMIM access and the name of the mt‐aaRS causing disease.
- The “Mutations Statistics” page contains all the statistics regarding any amino‐acid substitution for the 19 or individual mt‐aaRS. It compiles the number of missense and nonsense mutations, the conservation status of the residues affected by the mutation and the occurrence count of each amino‐acid substitution.
- The “Bibliography” page shows the list of related literature ordered chronologically and gives access to the publications through the PubMed Website.

### 2.3 Single mt‐aaRS window

To provide a unifying view and transversal analyses, each single mt‐aaRS window has four interactive tabs (*Integrative Analysis, Alignment, Bibliography*, and *Mutations Statistics*) and a ModMutGimmick. The ModMutGimmick shows the modular organization of the considered mt‐aaRS, with the mutations localized through colored lollipops that are connected according to allelic compositions in patients. Any action in one of the four tabs or on the ModMutGimmick is directly transferred and reflected on the other tabs.

**Figure 3:**
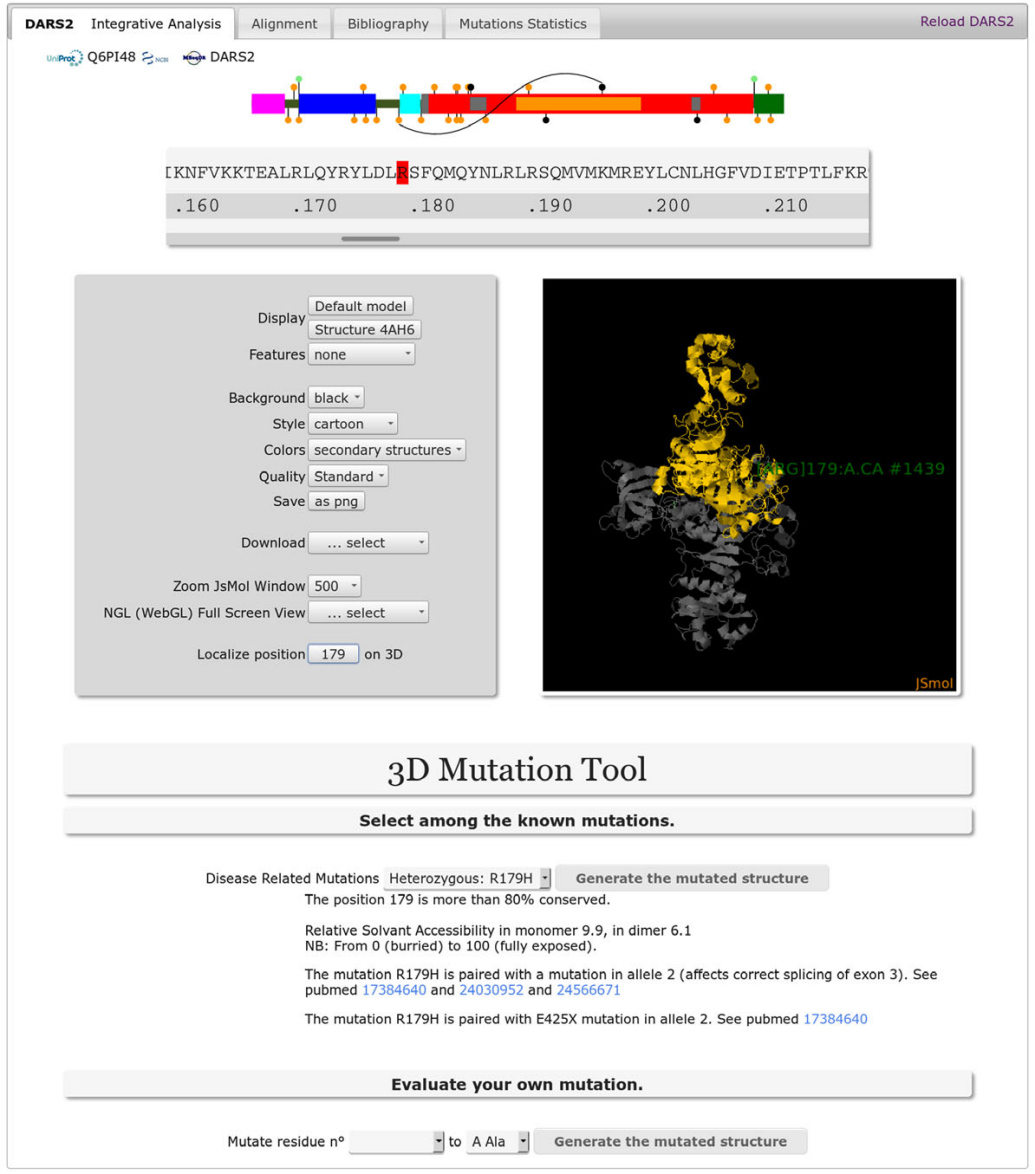
Screen capture of the DARS2 Integrative Analysis tab. From top to bottom, the page provides: UniProt/NCBI and MSeqDR links, the interactive ModMutGimmick with a black line indicating the compound heterozygous status of the patient, who has the R179H mutation associated with the E425X mutation, the linear sequence of the human mt‐AspRS, the JSmol 3D structure viewer window (either a crystallographic structure or a 3D model) with its control panel, the 3D Mutation toolbox to select and render the 3D model of a known disease‐related mutation or a user‐defined mutation. Upon selection of the R179H mutation in the dropdown menu, the conservation status, the relative solvent accessibility, and all known alleles with the bibliographic references are displayed. The mutated structure can be calculated and rendered by clicking on the “Generate the mutated structure” button. Finally, the same information can be obtained for a user‐defined mutation in the “Evaluate your own mutation” section

**Figure 4:**
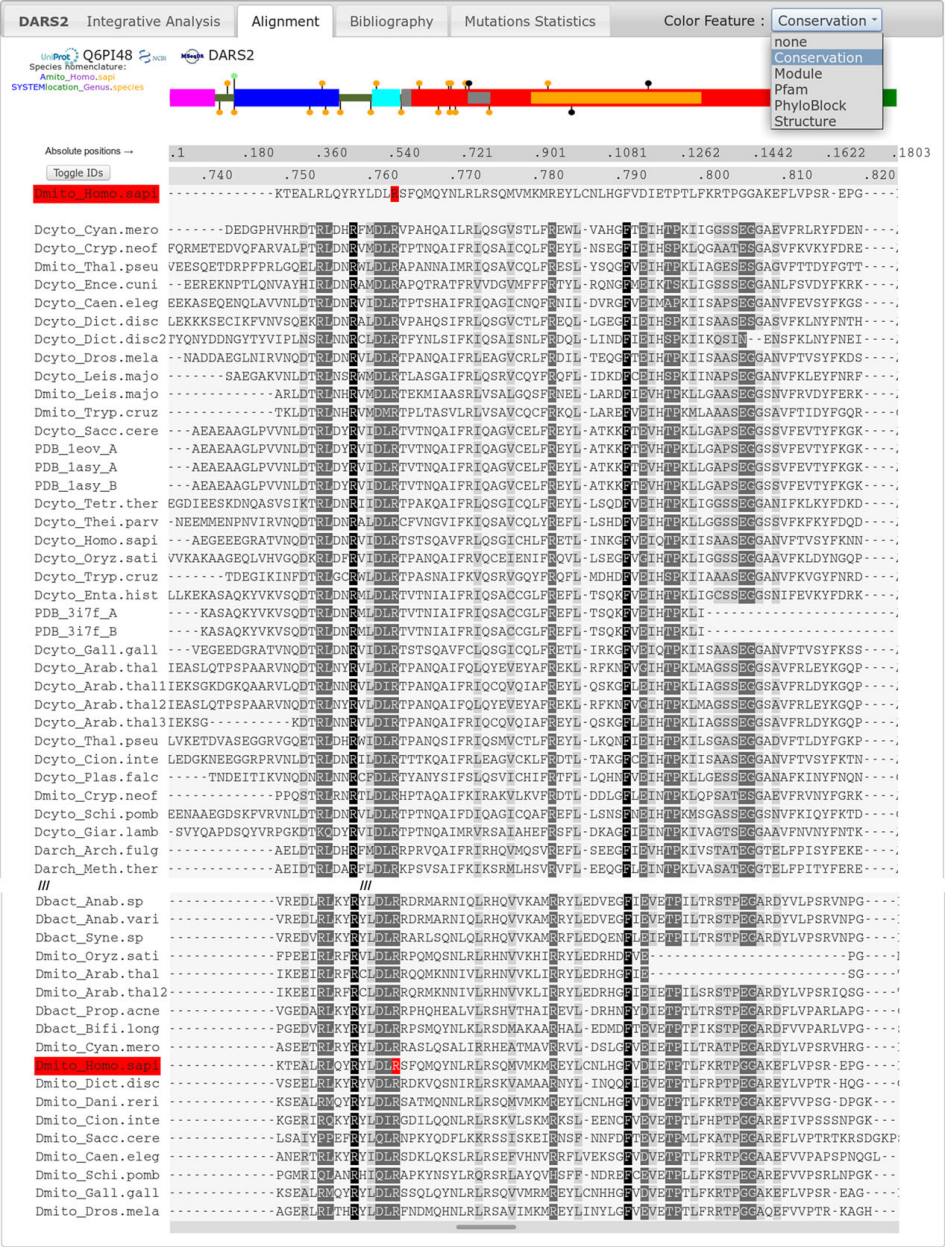
View of the DARS2 Alignment tab. Below the ModMutGimmick, the multiple sequence alignment of the mt‐AspRS sequences from bacterial, archaeal, and eukaryotic origin is visible through a sliding window. The human mt‐AspRS, highlighted in red, appears twice, at the top of the alignment and within its closest relatives. The Color Feature dropdown menu displays: (i) the conservation mode (black, gray, and light gray boxes correspond to 100% strictly conserved residues, more than 80% strictly conserved residue, and more than 60% physico‐chemically conserved residues, respectively); (ii) functional modules as shown in ModMutGimmick; (iii) PFAM domains; (iv) PhyloBlock; and (v) secondary structures. The absolute position ruler is scaled on the ModMutGimmick allowing a rapid focus to the clicked position. The “Toggle IDs” button switches between the default MiSynPat sequence name nomenclature and the UniProt/NCBI accession number. Clicking on one residue (indicated in red) of the human mt‐AspRS sequence from the alignment will automatically highlight this residue in all options of the Integrative Analysis tab

*The Integrative Analysis tab* includes the structural data to localize mutations on the sequence and 3D model, combined with the 3D Mutation toolbox to select and render the 3D model of a reported disease‐related or user‐defined mutation (Fig. 3). Users can visualize conservation, structural and clinical data and download high‐quality 3D images and PDB files of the wild type or of “on‐the‐fly” generated mutant molecules. *The Alignment tab* (Fig. 4) concerns the analysis of amino acid conservation based on a robust, structure‐based, and manually curated multiple sequence alignment of the aaRS sequences ranging from bacteria to human. The alignment can be colored according to five features (conservation, structural modules, Pfam, PhyloBlock, secondary structure). In addition, separate multiple sequence alignments focused on vertebrate species are provided. Finally, the relevant literature with general information and automatically compiled statistics are provided in *the Bibliography tab* and in *the Mutations tab*, respectively.

Disease‐related protein sequence variations are indicated within MiSynPat tabs following the HGVs international nomenclature standards (e.g., p.Arg179His, which indicates that Arginine at position 179 is replaced by an Histidine) and using the one letter code (e.g., R179H) in order to be easily recognizable within the multiple sequence alignments. It should be noted that help is provided in MiSynPat through tooltips when applicable, and a dedicated Help Page is accessible throughout the Website.

## 3 SURVEY OF DATA ANALYSIS

The automated follow‐up of disease‐related mt‐aaRS publications revealed that, just 10 years after the first description of patients with mutations in the *DARS2* gene (Scheper et al., 2007), each of the 19 human mt‐aaRS encoding genes has now been reported to contain disease‐causing mutations. Today, the total number of reported missense and nonsense mutations impacting coding sequences of mt‐aaRS genes amounts to 152 corresponding to 147 different positions. While patients are mostly compound heterozygotes, 26 of the 152 mutations are found in the homozygous state. The highest number of mutations is observed in *DARS2* (31 different mutations), followed by *EARS2* (22 mutations), *AARS2* (15 mutations) and *RARS2* (14 mutations). These genes were among the first to be correlated with human disorders (Schwenzer et al., 2014). There is a large panel of related autosomal recessive disorders, with reports of leukodystrophy (for mutations in *AARS2*, *DARS2*, *EARS2*, *MARS2*), epileptic encephalopathy (*CARS2*, *FARS2*, *PARS2*, *NARS2*, *IARS2*, *RARS2*, *TARS2*, *VARS2*), Perrault syndrome (*HARS2*, *LARS2*), MLASA syndrome (*YARS2*), HUPRA syndrome (*SARS2*), cardiomyopathy (*AARS2*, *KARS*, *GARS*), hearing loss or deafness (*MARS2*, *NARS2*), or intellectual disability (*RARS2*, *WARS2*). *GARS* and *KARS* represent more specific cases, in which reported dominant mutations are correlated with the Charcot‐Marie‐Tooth peripheral neuropathy. For these, the mt and the cytosolic GlyRSs and LysRSs are encoded by single genes and an impact of the mutations on the cytosolic function of the enzyme has been suggested (e.g., Diodato et al., 2014).

The highest number of mutations occurs in the catalytic domain (88 mutations) and in the anticodon‐binding domain (25 mutations), which are the two functional domains representing the major part of most of the mt‐aaRSs. However, no mutation has been observed in the strictly conserved catalytic residues from class I aaRSs (“KMSKS” and “HIGH”; Eriani, Delarue, Poch, Gangloff, & Moras, 1990). The 3D models for all the mt‐aaRSs and automatic tools to display the disease‐associated mutations on those models also shows that most of the mutations are not concentrated in any specific spatial region, and are not differentially distributed between buried or solvent accessible positions. Importantly, 96 of the 147 positions affected by disease‐associated mutations are not conserved throughout the sequences covering the phylogeny, and only nine of the remaining 51 positions are 100% evolutionarily conserved.

## 4 MATERIAL AND METHODS

### 4.1 Database architecture

The database (see Supp. Fig. S1) consists of four main tables called *Synthetase*, *Disease*, *Mutation*, and *Bibliography*. Two junction tables, ln_biblio_synthetase and ln_disease_synthetase, link each Synthetase data to its corresponding Bibliography and Disease data. The Synthetase table contains standard information about each protein (gene name, protein name, UniProt accession number, date of last update, class I or II, and the mutation and structural features). The Disease table provides name, acronym, and OMIM entry number. The Mutation table contains the foreign keys linking to the associated Disease, Synthetase, and Bibliography as well as the variations in both alleles and a comment. The Bibliography table orders the publications as they are incorporated and provides the PMID and information on the abstract, title, journal, and publication date.

### 4.2 Sequence alignment, features, and conservation

For each aminoacylation system, a manually curated 3D structure‐guided multiple sequence alignment has been built using sequences from 92 complete genome organisms representative of bacterial, archaeal and eukaryotic phylogenetic diversity (see Supp. Table S1). In addition, 19 manually curated multiple sequence alignments of vertebrate mt‐aaRS have been built using sequences from 58 high‐quality UniProt reference proteomes representative of the Vertebrate diversity (see Supp. Table S2).

Each sequence is identified by an in‐house defined MiSynPat sequence nomenclature composed of: a one letter code of the aaRS system followed by a four letter code indicating the Bacterial (bact), Archaeal (arch), or Eukaryotic cellular location (cyto for cytosolic, mito for mitochondrial, chlo for chloroplastic), then an underscore sign followed by 2 four letter codes for the Genus and species separated by a dot (e.g., Dmito_Homo.sapi stands for the human DARS2).

Each alignment has been treated by the MACSIMS program (Thompson et al., 2006) in order to collect and propagate associated functional/structural features (PFAM‐A domains, PhyloBlock, secondary structures). We defined three conservation levels: residues with 100% identity over a column, residues with >80% identity over a column and columns with >60% of similar residues using the six similar residue groups [(I, L, M,V); (P, A, G, S, T); (F, Y, W); (K, R, H); (D, E, Q, N); and C].

### 4.3 Structural information

To date, only three human mt‐aaRS crystallographic structures have been solved [mt‐TyrRS (Bonnefond et al., 2007), mt‐PheRS (Klipcan et al., 2012), and mt‐AspRS (Neuenfeldt et al., 2013)]. Models of all other human mt‐aaRS are built using the PHYRE2 Web server with the "intensive" option (Kelley, Mezulis, Yates, Wass, & Sternberg, 2015). Using the PDB (Berman et al., 2000) RCSB Web services, an automatic weekly search retrieves all aaRS structures and all human mt‐aaRS structures. If a new structure of a human mt‐aaRS is published, the calculated model is replaced. If a non‐human related structure is released in the PDB, a new PHYRE2 model is calculated. When applicable, the LSQMAN program (Kleywegt & Jones, 1994) is used to build the dimeric mt‐aaRS by superposing the computed monomer over the two chains of the closest dimer. The model of a given mutation is computed by the Modeler script program using default parameters (Fiser & Sali, 2003). The protein residue Relative Solvent Accessibility (RSA) is computed with the NAccess program (Hubbard & Thornton, 1993), using a default probe radius of 1.4 Å and a standard Ala‐XXX‐Ala tripeptide as the full accessibility reference for amino acid XXX.

### 4.4 Additional dataset links and tools

Links to MSeqDR (https://mseqdr.org/), OMIM (https://www.omim.org), UniProt (http://www.uniprot.org/), NCBI (https://www.ncbi.nlm.nih.gov/), RSCB PDB (http://www.rcsb.org/pdb/home/home.do), and PubMed (https://www.ncbi.nlm.nih.gov/pubmed/) are provided by clicking on the relevant IDs when applicable throughout MiSynPat. The PolyPhen (Adzhubei et al., 2010) and SIFT (Sim et al., 2012) predictions for a given mutation are computed on the fly within the Integrative Analysis tab.

### 4.5 Bibliographic information

Articles concerning human mt‐aaRS diseases are automatically retrieved from PubMed at the NCBI through the E‐utilities Web service, using a filter built from a training set composed of 102 relevant articles published from 2007 and 2014. The first sieve of the filter consists of a set of relevant terms linked by Boolean operators to query the NCBI PubMed fields: Title/Abstract and Text Word. The coarseness of the filter has been adjusted so that no article of the training set is rejected. The selected articles are presented through a Web form to an expert who validates or rejects the proposed articles and enters the relevant information regarding all types of mutations (missense, nonsense, insertion, deletion, splicing defect) or diseases into the database. The bibliography search and validation process is performed daily and has been tested over a period of 18 months.

### 4.6 Implementation

All MiSynPat SQL tables are implemented in a SQLite3 database (v3.8.8.3). The Web server is written in Tcl (v 8.6.4) hosted by an Apache server (v2.4) running the Rivet (v2.2) module under Ubuntu. The client side in written in HTML5/jQuery 1.11 and uses the JSmol plugin (an open‐source HTML5 viewer for chemical structures in 3D; http://wiki.jmol.org/index.php/JSmol).

## 5 CONCLUSIONS

The main aim of MiSynPat is to help to bridge the gap between clinicians and basic researchers working on human mt‐aaRS‐related disorders. The diseases linked to mutations in the nuclear genes coding for mt‐aaRSs are now described at an increasing frequency and revealed to be complex. The complexity is not only due to the variety of phenotypic expressions, with tissue‐specific phenotypic imprints, but also due to the absence, for the moment, of clear mechanistic explanations for most of the systems. In MySinPat, mutations discovered in the past ten years are now enriched by weekly reports. MiSynPat, by allying sequence data, allelic composition in patients, sequence conservation, structural information, and bibliography, offers a homogeneous resource for in‐depth analyses. The conservation/structural standpoint perfectly complements existing databases, whose main goal is to facilitate genomic investigation (e.g., MSeqDR; Shen et al., 2016). In addition, the embedded infrastructure and the automated mining and retrieval of literature data with minimal human intervention guarantee durability, regularity and up‐to‐date maintenance of the site.

The unique configuration of MiSynPat explains why being restricted to a family of enzymes is a true added value and allows users a more comprehensive view of the field. Indeed, aaRSs, as a family of ancillary/housekeeping enzymes, have been studied structurally and functionally for decades. We now have a broad understanding of these enzymes (e.g., Bullwinkle & Ibba, 2014; Giegé & Springer, 2016; Havrylenko & Mirande, 2015; Schimmel, Giegé, Moras, & Yokoyama, 1993). For instance, crystallographic structures are available for homologous proteins that are representative of all aaRSs. This allowed us not only to provide structural alignments (based on structural data), but also to generate 3D models for any of the human mt‐aaRSs, and thus for any of the mutated mt‐aaRSs. In‐depth investigations and knowledge also clearly identified substrate interacting interfaces and uncovered, at least for cytosolic aaRSs, a large number of non‐canonical functions, beyond translation (e.g., Guo & Schimmel, 2013; Guo, Yang, & Schimmel, 2010; Kim, You, & Hwang, 2011; Ray, Arif, & Fox, 2007). It is only recently that a similar example has been identified for the rat mt‐TrpRS (Wang et al., 2016), opening the doors for alternate roles of mt‐aaRSs.

Currently, the global view of disease‐associated mutations impacting mt‐aaRSs excludes a simple mechanistic explanation, and instead suggests that analysis of multiple mutations at once, rather than a one‐at‐a‐time approach, will lead to a better understanding of the associated diseases. MiSynPat thus offers a way to categorize the mutations in the affected mt‐aaRSs, and to distinguish those that may interrupt functions affecting protein synthesis from those that may disrupt alternate function(s) (at, e.g., non‐conserved position(s) and/or enzyme surfaces that do not interact with tRNA), which appeared in mammals and are not directly involved in protein synthesis. As such, the body of knowledge provided by MiSynPat will form the core of a future expert system further bridging the gap between clinical data and mechanistic interpretations.

## Supporting information

Supporting Material
