# Supplementary material for: MiSynPat: An integrated knowledge base linking clinical, genetic, and structural data for disease‐causing mutations in human mitochondrial aminoacyl‐tRNA synthetases
Source: Hum Mutat. 2017 Jun 27;38(10):1316–24. doi: 10.1002/humu.23277 (PMC5638098; doi:10.1002/humu.23277)
Supplement: Supplementary file 1 — Supporting Material [file HUMU-38-1316-s001.pdf]

SUPPLEMENTARY MATERIAL

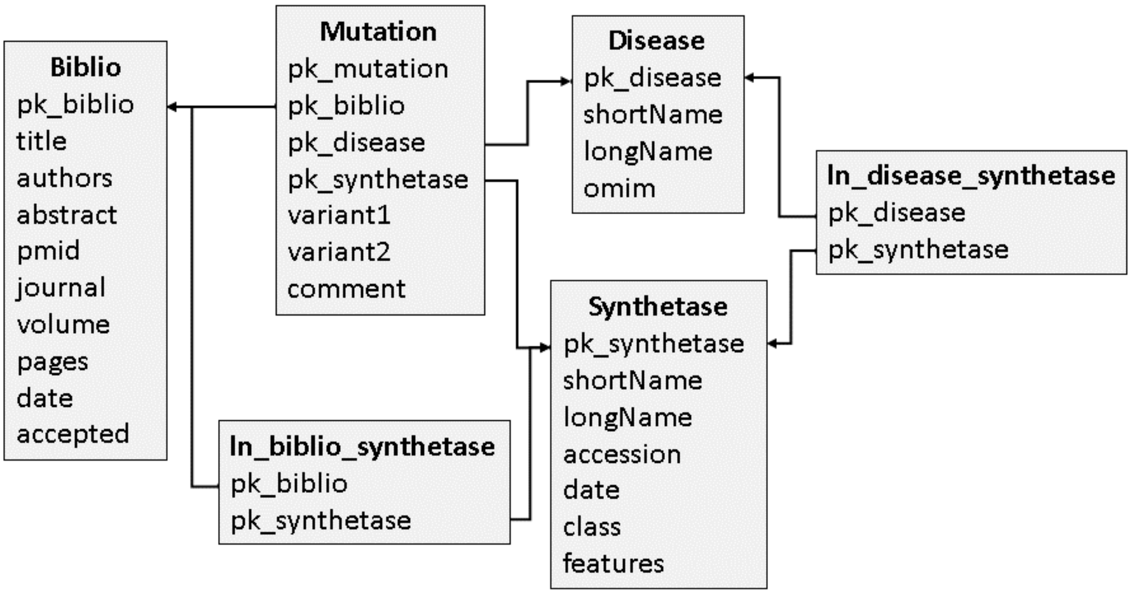

Supplementary Figure S1: Schematic presentation of the overall architecture and organization of the MiSynPat Sql Database Schema

Supplementary Table S1: List of the 93 organisms queried to build the manually curated 3D structure-guided multiple sequence alignments. Names of organisms are classified according to the belonging to alpha-proteobacteria, cyanobacteria, bacteria, archaea and eukarya. Taxonomy ID (NCBI) / in-house defined MiSynPat sequence nomenclature / species name (binomiale nomenclature)

alpha-proteobacteria
181661 / Agro.tume / Agrobacterium tumefaciens
770 / Anap.marg / Anaplasma marginale
38323 / Bart.hens / Bartonella henselae
803 / Bart.quin / Bartonella quintana
375 / Brad.japo / Bradyrhizobium japonicum
235 / Bruc.abor / Brucella abortus
29459 / Bruc.meli / Brucella melitensis
29461 / Bruc.suis / Brucella suis
335992 / Cand.Pela / Candidatus Pelagibacter ubique
155892 / Caul.cres / Caulobacter crescentus
944 / Ehrl.cani / Ehrlichia canis
302409 / Ehrl.rumi / Ehrlichia ruminantium Gardel
254945 / Ehrl.rumi / Ehrlichia ruminantium Welgevonden
442 / Gluc.oxyd / Gluconobacter oxydans
381/ Meso.loti / Mesorhizobium loti
1076 / Rhod.palu / Rhodopseudomonas palustris
781 / Rick.cono / Rickettsia conorii
42862 / Rick.feli / Rickettsia felis
782 / Rick.prow / Rickettsia prowazekii
785 / Rick.typh / Rickettsia typhi
89184 / Sili.pome / Silicibacter pomeroyi
382 / Sino.meli / Sinorhizobium meliloti
292805 / Wolb.endo / Wolbachia endosymbiont of B. malayi
163164 / Wolb.endo / Wolbachia endosymbiont of D. melano
542 / Zymo.mobi / Zymomonas mobilis

cyanobacteria
1172 / Anab.vari / Anabaena variabilis
1177 / Anab.sp / Nostoc
1142 / Syne.sp / Synechocystis

bacteria
63363 / Aqui.aeol / Aquifex aeolicus
29544 / Azoa.sp. / Azoarcus sp.
1392 / Baci.anth / Bacillus anthracis
818 / Bact.thet / Bacteroides thetaiotaomicron
216816 / Bifi.long / Bifidobacterium longum
139 / Borr.burg / Borreliella burgdorferi
197 / Camp.jeju / Campylobacter jejuni
129958 / Carb.hydr / Carboxydothermus hydrogenoformans

83560 / Chla.muri / Chlamydia muridarum
83558 / Chla.pneu / Chlamydia pneumoniae
1097 / Chlo.tepi / Chlorobaculum tepidum
61435 / Deha.ethe / Dehalococcoides ethenogenes
1299 / Dein.radi / Deinococcus radiodurans
562 / Esch.coli / Escherichia coli
851 / Fuso.nucl / Fusobacterium nucleatum
35554 / Geob.sulf / Geobacter sulfurreducens
33959 / Lact.john / Lactobacillus johnsonii
1575 / Leif.xyli / Leifsonia xyli
173 / Lept.inte / Leptospira interrogans
28227 / Myco.pene / Mycoplasma penetrans
487 / Neis.meni / Neisseria meningitidis
1747 / Prop.acne / Propionibacterium acnes
265606 / Rhod.balt / Rhodopirellula baltica
1280 / Stap.aure / Staphylococcus aureus
1313 / Stre.pneu / Streptococcus pneumoniae
1143 / Syne.sp. / Synechocystis sp.
2336 / Ther.mari / Thermotoga maritima
2039 / Trop.whip / Tropheryma whipplei
670 / Vibr.para / Vibrio parahaemolyticus

archae
56636 / Aero.pern / Aeropyrum pernix
2234 / Arch.fulg / Archaeoglobus fulgidus
2243 / Halo.sp. / Halobacterium sp.
145262 / Meth.ther / Methanothermobacter thermautotrophicus
2190 / Meth.jann / Methanocaldococcus jannaschii
2320 / Meth.kand / Methanopyrus kandleri
2209 / Meth.maze / Methanosarcina mazei
160232 / Nano.equi / Nanoarchaeum equitans
13773 / Pyro.aero / Pyrobaculum aerophilum
2261 / Pyro.furi / Pyrococcus furiosus
2287 / Sulf.solf / Sulfolobus solfataricus
2303 / Ther.acid / Thermoplasma acidophilum

eukaryota
3702 / Arab.thal / Arabidopsis thaliana
7955 / Dani.reri / Danio rerio
6239 / Caen.eleg / Caenorhabditis elegans
7719 / Cion.inte / Ciona intestinalis
5207 / Cryp.neof / Cryptococcus neoformans
45157 / Cyan.mero / Cyanidioschyzon merolae
44689 / Dict.disc / Dictyostelium discoideum
7227 / Dros.mela / Drosophila melanogaster
6035 / Ence.cuni / Encephalitozoon cuniculi
5759 / Enta.hist / Entamoeba histolytica
9031 / Gall.gall / Gallus gallus

5741 / Giar.inte / Giardia intestinalis
9606 / Homo.sapi / Homo sapiens
5664 / Leis.majo / Leishmania major
4530 / Oryz.sati / Oryza sativa
5833 / Plas.falc / Plasmodium falciparum
4932 / Sacc.cere / Saccharomyces cerevisiae
4896 / Schi.pomb / Schizosaccharomyces pombe
7668 / Stro.purp / Strongylocentrotus purpuratus
5911 / Tetr.ther / Tetrahymena thermophila
35128 / Thal.pseu / Thalassiosira pseudonana
5875 / Thei.parv / Theileria parva
5722 / Tric.vagi / Trichomonas vaginalis
5693 / Tryp.cruz / Trypanosoma cruzi

Supplementary Table S2: List of the 58 organisms queried to build the vertebrate manually curated multiple sequence alignments. Taxonomy ID (NCBI) / in-house defined MiSynPat sequence nomenclature / species name (binomiale nomenclature)

9646 / Ailu.mela / Ailuropoda melanoleuca
8496 / Alli.miss / Alligator mississippiensis
12930 / Amaz.aest / Amazona aestiva
28377 / Anol.caro / Anolis carolinensis
7994 / Asty.mexi / Astyanax mexicanus
9913 / Bos.taur / Bos taurus
9483 / Call.jacc / Callithrix jacchus
419612 / Came.feru / Camelus ferus
9612 / Cani.lupu / Canis lupus
10141 / Cavi.porc / Cavia porcellus
8469 / Chel.myda / Chelonia mydas
60711 / Chlo.saba / Chlorocebus sabaeus
7955 / Dani.reri / Danio rerio
9796 / Equu.caba / Equus caballus
9685 / Feli.catu / Felis catus
59894 / Fice.albi / Ficedula albicollis
885580 / Fuko.dama / Fukomys damarensis
9031 / Gall.gall / Gallus gallus
69293 / Gast.acul / Gasterosteus aculeatus
9593 / Gori.gori / Gorilla gorilla
10181 / Hete.glab / Heterocephalus glaber
9606 / Homo.sapi / Homo sapiens
43179 / Icti.trid / Ictidomys tridecemlineatus
215358 / Lari.croc / Larimichthys crocea
7897 / Lati.chal / Latimeria chalumnae
7918 / Lepi.ocul / Lepisosteus oculatus
9785 / Loxo.afri / Loxodonta africana
9541 / Maca.fasc / Macaca fascicularis
9544 / Maca.mula / Macaca mulatta
13616 / Mono.dome / Monodelphis domestica
10090 / Mus.musc / Mus musculus
9668 / Must.puto / Mustela putorius
109478 / Myot.bran / Myotis brandtii
225400 / Myot.davi / Myotis davidii
59463 / Myot.luci / Myotis lucifugus
61853 / Noma.leuc / Nomascus leucogenys
8665 / Ophi.hann / Ophiophagus hannah
8128 / Oreo.nilo / Oreochromis niloticus
9258 / Orni.anat / Ornithorhynchus anatinus
9986 / Oryc.cuni / Oryctolagus cuniculus
8090 / Oryz.lati / Oryzias latipes
30611 / Otol.garn / Otolemur garnettii
9940 / Ovis.arie / Ovis aries
9598 / Pan.trog / Pan troglodytes

9555 / Papi.anub / Papio anubis
13735 / Pelo.sine / Pelodiscus sinensis
48698 / Poec.form / Poecilia formosa
9601 / Pong.abel / Pongo abelii
9402 / Pter.alec / Pteropus alecto
10116 / Ratt.norv / Rattus norvegicus
9305 / Sarc.harr / Sarcophilus harrisii
113540 / Scle.form / Scleropages formosus
9823 / Sus.scro / Sus scrofa
31033 / Taki.rubr / Takifugu rubripes
99883 / Tetr.nigr / Tetraodon nigroviridis
246437 / Tupa.chin / Tupaia chinensis
8364 / Xeno.trop / Xenopus tropicalis
8083 / Xiph.macu / Xiphophorus maculatus

Supplementary Table S3: Color code used for aaRS structural domains and lollipops representing the disease-related mutations

Color	aaRS structural domains
green	alpha-helical domain
blue	anticodon-binding domain
green	bacterial-type C-terminal extension
brown	C-terminal extension domain
red	catalytic domain
cyan	helix of dimerization
gray	HIGH (class I aaRS catalytic residues)
orange	insertion domain
gray	KMSKS (class I aaRS catalytic residues)
gray	motif 1 (Class II aaRS catalytic residues)
gray	motif 2 (Class II aaRS catalytic residues)
gray	motif 3 (Class II aaRS catalytic residues)
magenta	putative mitochondrial targeting sequence (MTS)
light green	SAD Domain (ThrRS and AlaRS Second Additional Domain)
green	TGS domain (beta-sheet structure specific from threonyl-tRNA synthetase (ThrRS), GTPase, and guanosine-3',5'-bis(diphosphate) 3'-pyrophosphohydrolase (SpoT)
green	tRNA edge-binding domain
green	WHEP domain (Helix-turn-helix domain specific from TrpRS, HisRS, GluProRS)

Color	lollipops (disease-related mutations)
cyan	missense dominant mutation (heterozygous)
orange	missense recessive mutation (compound heterozygous)
black	nonsense recessive mutation (compound heterozygous)
green	missense recessive mutation (homozygous)
